# ECCM Scheme against Interrupted Sampling Repeater Jammer Based on Parameter-Adjusted Waveform Design

**DOI:** 10.3390/s18041141

**Published:** 2018-04-08

**Authors:** Zhenhua Wei, Zhen Liu, Bo Peng, Rui Shen

**Affiliations:** 1 Rocket Force University of Engineering, Xi’an 710025, China; wzh016001@aliyun.com; 2 College of Electronic Science, National University of Defense Technology, Changsha 410073, China; pengbo06@gmail.com (B.P.); sr_neu@163.com (R.S.)

**Keywords:** interrupted sampling repeater jamming (ISRJ), waveform design, electronic counter-counter measure (ECCM), time-frequency (TF) analysis

## Abstract

Interrupted sampling repeater jamming (ISRJ) is an effective way of deceiving coherent radar sensors, especially for linear frequency modulated (LFM) radar. In this paper, for a simplified scenario with a single jammer, we propose a dynamic electronic counter-counter measure (ECCM) scheme based on jammer parameter estimation and transmitted signal design. Firstly, the LFM waveform is transmitted to estimate the main jamming parameters by investigating the discontinuousness of the ISRJ’s time-frequency (TF) characteristics. Then, a parameter-adjusted intra-pulse frequency coded signal, whose ISRJ signal after matched filtering only forms a single false target, is designed adaptively according to the estimated parameters, i.e., sampling interval, sampling duration and repeater times. Ultimately, for typical jamming scenes with different jamming signal ratio (JSR) and duty cycle, we propose two particular ISRJ suppression approaches. Simulation results validate the effective performance of the proposed scheme for countering the ISRJ, and the trade-off relationship between the two approaches is demonstrated.

## 1. Introduction

Interrupted sampling repeater jamming (ISRJ) is a recently-developed, efficient, active jamming method [1,2,3] for radar sensors. Through low-rate interrupted sub-sampling, ISRJ is able to produce a series of coherent false targets in the pulse compression result of the radar receiver without sampling and storing the whole radar pulse, in contrast to traditional jamming approaches. Furthermore, this method has great advantages due to its adoption of the receive-transmit time-sharing antenna system, which significantly ameliorates the response speed and analog-to-digital (AD) sampling rate of the jammer. As ISRJ exhibits excellent performance, with both deceptive and blanketing characteristics [4,5,6], corresponding anti-jamming methods (i.e., jamming recognition and jamming suppression) need to be developed for self-defense.

Up until now, there have been two main directions in electronic counter-counter measure (ECCM) research on countering ISRJ. On the one hand, based on the traditional liner modulated frequency (LMF) radar, there has been sporadic research about ECCM for ISRJ, most of which focuses on the radar receiver to develop signal processing and jamming recognition methods. In [7], by performing time-frequency (TF) analysis on the radar echo signal containing ISRJ, the discontinuous characteristics of the ISRJ in the TF domain are exploited to determine the jamming zone of the echo signal, which is eliminated to obtain the pulse compression result of the target only. However, since the frequency continuity of the target echo signal is also destroyed, this method suffers from high side-lobes in the pulse compression result, which decrease the target detection performance. On the other hand, various signal waveforms definitely have different anti-jamming abilities, and several authors have analyzed the performance of ISRJ with different radar signals [8,9,10], with their research mainly investigating the influence of different radar waveform parameters on jamming effect. For example, Letao Xu (2012) analyzed the ISRJ effect on phase coded (PC) signal in [11] and pointed out that ISRJ is not sensitive to the coding mode of the phase coded signal, and can only form a single false target behind the real target. Rui Shen (2016) highlighted that an efficient jamming effect on an intra-pulse frequency coded (IPFC) signal [12] can be achieved only using the periodic repeater method with appropriate parameters [13]. The above references indicate that some inner-pulse modulated signals with appropriate parameters may have preferable anti-jamming performance compared to the LMF signal, because the corresponding ISRJ signals, after matched filtering, always form a single false target. However, to the best knowledge of the authors, ECCM research against ISRJ based on signal design has not been reported. Therefore, the suppression of ISRJ, especially via radar waveform design, needs to be further developed.

In this paper, a cognitive ECCM scheme is adopted to counter ISRJ for a simplified scenario with a single jammer. In the scheme, we first transmit the LFM signal to estimate the main jamming parameters by exploiting the discontinuous characteristics of ISRJ in the TF domain, and then a parameter-adjusted IPFC signal is designed adaptively. Then, for typical jamming scenes with different jamming signal ratio (JSR) and duty cycle, we propose two ISRJ suppression approaches based on the designed signal, i.e., band-pass filter design method (Approach I), and signal shearing and splicing method (Approach II). Approach I generates a time-domain band-pass filter that can repress the power of the false target effectively; however, the false target cannot be totally removed, and the suppression performance will be decreased when the jamming signal ratio (JSR) is large. Thus, at the cost of sacrificing the signal-to-noise ratio (SNR), Approach II eliminates the echo in the jamming zone and splices the rest of them, to retain the true target and suppress the ISRJ. Finally, the selection principle between the two approaches is analyzed in detail.

The remainder of this paper is organized as follow. Section 2 reviews the principle of ISRJ and investigates the differences between the target signal and ISRJ signal in the TF domain. Moreover, the execution steps of ISRJ parameter estimation are proposed. In Section 3, we introduce our two proposed ECCM approaches, and the selection principle between the two approaches under the whole ECCM scheme is described explicitly. Section 4 carries out some detailed experimental results to demonstrate the superiority of our framework. Concluding remarks and directions for future research are presented in Section 5.

## 2. Signal Modeling

In this section, firstly, we review the generally applicable theory of ISRJ, which is the foundation of the research work in this paper. Subsequently, based on the LMF signal model, we analyze the TF characteristics by performing the short-time Fourier transform (STFT) on the received radar signal to find the difference between the target signal and ISRJ. This is evidently to detect whether the ISRJ signal is discontinuous and periodic in the TF domain, and we can thus estimate the key parameters of the ISRJ, including pulse repeat interval, sampling duration and repeater times, by adopting correlation detection algorithms. Ultimately, execution steps for an ISRJ parameter estimation method based on TF analysis are proposed.

### 2.1. The Principle of ISRJ

**Figure 1:**
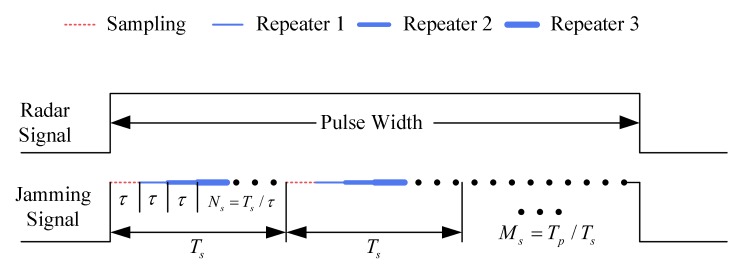
Diagram of the ISRJ method.

In the ISRJ method, parts of the radar signal are firstly sampled to store in memory. Next, the jammer transmits the stored signal repeatedly to counter the radar. A diagram of the ISRJ method is shown in Figure 1. As [4] noted that the interrupted sampling function p(t) is defined as

(1) $$p(t)=\mathrm{rect}\left(\frac{t}{\tau }\right)⊗\sum_{-\infty }^{+\infty }\delta (t-nT_{s})$$

where τ is the sampling duration, $\mathrm{rect}\left(\frac{t}{\tau }\right)=1$ for 0≤t≤τ and zeros otherwise, ⊗ represents the convolution operation, $T_{s}$ is the pulse repeat interval, $k=\frac{\tau }{T_{s}}$ denotes the sampling duty cycle.

Assume that the radar signal is x(t) with pulse duration *T* and bandwidth *B*. The interrupted samples of the radar signal are equivalent to x(t) multiplied with p(t), so the sampled signal $x_{s}(t)$ is given as

(2) $$x_{s}(t)=p(t)x(t)$$

After continuous delay, the sampled signal is transmitted repeatedly and the ISRJ is ultimately generated, and can be expressed as

(3) $$x_{J}(t)=A_{J}\sum_{m=1}^{N_{s}}x_{s}(t-m\tau )=A_{J}\sum_{m=1}^{N_{s}}p(t-m\tau )x(t-m\tau )$$

where the τ is the sampling duration, $N_{s}$ denotes the repeater times, and $A_{J}$ represents the transmitting amplitude gain.

### 2.2. TF Analysis for Target Signal and ISRJ Signal

TF transform and analysis, such as STFT [14], is commonly applied to analyze the TF characteristics of the general signal. However, it is difficult to obtain a certain mathematical expression of the TF transform result. For certain signals with only one frequency component at all times, the TF characteristics can be denoted explicitly by their instantaneous frequency (IF), which is the derivative of the signal phase [15]. Therefore, here we use IF to mathematically analyze the TF characteristics of the target signal and ISRJ signal. It should be stated that, in practical procedures, as well as simulations, STFT is still applied to obtain the TF results, and is actually a typical method of IF estimation. 

According to Section 2, we assume that radar transmits the LMF signal. Without loss of accuracy of TF analysis, we could ignore the propagation distance between radar and target to simplify the target signal. Therefore, the IF of the target signal is

(4) $$f_{T}(t)=\mathrm{rect}\left(\frac{t}{T_{p}}\right)\cdot \frac{d}{dt}\left(f_{c}\hat{t}+\frac{1}{2}\pi \gamma t^{2}\right)=\mathrm{rect}\left(\frac{t}{T_{p}}\right)\cdot \left(f_{c}+\pi \gamma t\right)$$

Further, we can get the IF of the ISRJ signal as follows.

(5) $$\begin{array}{cc} f_{J}(t) & =\sum_{m=1}^{N_{s}}\mathrm{rect}\left(\frac{t-m\tau }{\tau }\right)\frac{d}{dt}\left[f_{c}(t-m\tau )+\frac{1}{2}\gamma {\left(t-m\tau \right)}^{2}\right]\\ & =\sum_{m=1}^{N_{s}}\mathrm{rect}\left(\frac{t-m\tau }{\tau }\right)\left[f_{c}+\gamma (t-m\tau )\right] \end{array}$$

**Figure 2:**
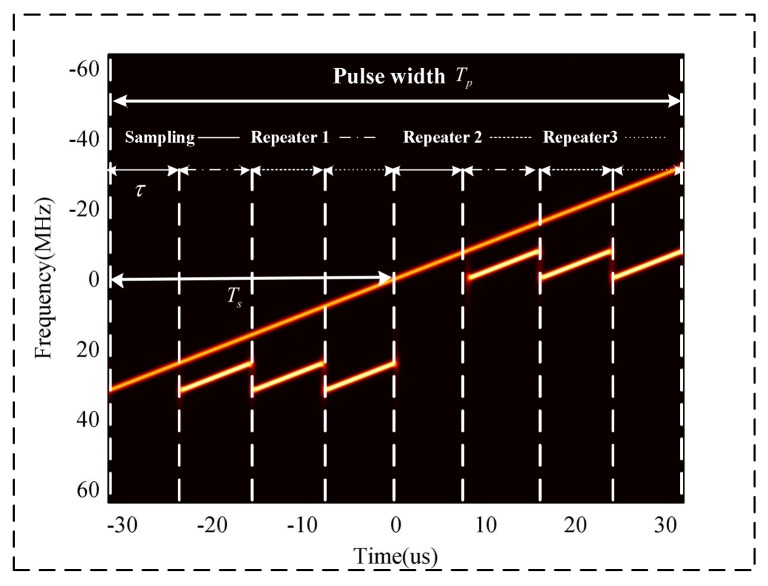
TF characteristics of LMF echo signal.

From (4) and (5), we can see that the relationships between the frequency and time for the ISRJ signal are similar to those of the target. Thus, the ISRJ can form a group of false targets in the pulse compression results of a matched filter. However, because of $m\tau <T_{s}$, the IF characteristics of the ISRJ are discontinuous in terms of pulse duration. Figure 2 shows the TF characteristics of radar echo signal, which contains the target signal and ISRJ by performing STFT. From Figure 2, it can be clearly seen that the frequency components of the target signal are continuously changing with the time of the pulse duration, and the IF characteristics of the ISRJ signal are discontinuous in terms of pulse duration because the receive-transmit antenna of ISRJ jammer must work asynchronously, and jamming signals are not transmitted during the duration in which the jammer is receiving the radar signal.

### 2.3. Executing Steps of ISRJ Parameter Estimation

Considering the practical situation, we could just obtain the radar echo signal comprised of target signal and jamming signal. In the traditional estimation method, jamming signal is always distinguished out of the radar signal for estimating its parameters. In this paper, based on the discontinuous and periodic characteristics of the ISRJ in the TF domain, we mainly use the STFT result of the radar echo signal for the ISRJ parameter estimation by calculating the correlation value of corresponding sequences and adopting the peak detection algorithm. The specific steps can be expressed as follows.

Step 1: By performing STFT, we can attain the TF characteristics of radar echo signal $x_{r}(t)$, which can be described as

(6) $$S(t,f)=\int_{-\infty }^{\infty }x_{r}(t^{\prime })\omega (t^{\prime }-t)\exp(-j2\pi ft^{\prime })dt^{\prime }$$

where ω(t) is the frequency smoothing window of the STFT.

Step 2: Based on the calculated STFT result S(t,f), we cumulate |S(t,f)| along the frequency to obtain the expression S(t), which could represent the power of radar echo signal.

(7) $$S(t)=\sum_{f}\left|S(t,f)\right|$$

Step 3: From S(t), we can to a significant degree find the periodicity of the radar echo signal, which is mainly influenced by the ISRJ signal, which appears periodically. Thus, after calculating the autocorrelation result of S(t) as (8), we can estimate the jammer repeat sampling interval ${\hat{T}}_{s}$ by measuring the distance between the extreme points of S(t)’s autocorrelation function.

(8) $${\langle S(t^{\prime })S^{*}(t^{\prime }+t)\rangle }_{t_{p}}=\frac{1}{t_{p}}\int_{t^{\prime }=0}^{t_{p}}S(t^{\prime })S^{*}(t^{\prime }+t)dt^{\prime }$$

Based on the periodicity and the discontinuous characteristics of S(t), the interrupted sampling function $\hat{p}(t)$ is reconstructed. By multiplying the transmitted radar signal x(t) with the interrupted sampling function $\hat{p}(t)$, the interrupted sampled signal can be attained as

(9) $${\hat{x}}_{s}(t)=\hat{p}(t)x(t)$$

Step 4: After calculating the cross-correlation result of interrupted sampled signal ${\hat{x}}_{s}(t)$ with radar echo signal $x_{r}(t)$, we can estimate the sampling duration $\hat{\tau }$ and the repeater times ${\hat{N}}_{s}$ by measuring the distance and the number of the extreme point of cross-correlation function obtained by (10).

(10) $${\langle {\hat{x}}_{s}(t^{\prime })x_{r}^{*}(t^{\prime }+t)\rangle }_{t_{p}}=\frac{1}{t_{p}}\int_{t^{\prime }=0}^{t_{p}}{\hat{x}}_{s}(t^{\prime })x_{r}^{*}(t^{\prime }+t)dt^{\prime }$$

Thus, by calculating the correlation value of the corresponding sequences, we can obtain the ISRJ parameter estimation results precisely based on the discontinuous and periodic characteristics of the ISRJ signal.

Then, the sampling duty cycle $\hat{k}$ can be estimated as

(11) $$\hat{k}=\frac{\hat{\tau }}{{\hat{T}}_{s}}$$

We assume that the distribution of signal power during pulse duration is uniform. Based on the above estimated parameters, the JSR can be derived as

(12) $$J\hat{S}R=\frac{P_{J}}{P_{S}}=\frac{\int_{\hat{\tau }}^{\hat{\tau }+{\hat{N}}_{s}\cdot \hat{\tau }}{\left[x_{r}(t)\right]}^{2}dt-{\hat{N}}_{s}\cdot \int_{0}^{\hat{\tau }}{\left[x_{r}(t)\right]}^{2}dt}{\int_{0}^{\hat{\tau }}{\left[x_{r}(t)\right]}^{2}dt}\cdot \hat{k}$$

## 3. ECCM Scheme against the ISRJ

In [13], we declared that the intra-pulse frequency coded signal has significant performance for ECCM, which only forms a single false target after matched filtering with proper parameter setting. We thus firstly analyze the influence on ECCM performance with different waveform parameters, and then a parameter-adjusted IPFC waveform is designed based on the estimated parameters above.

### 3.1. Transmission Waveform Optimization Design

The complex envelope of a IPFC signal whose hopping sequence is

(13) $$a=\left\{a_{1},a_{2},\ldots ,a_{M}\right\}$$

can be described as

(14) $$x(t)=\frac{1}{\sqrt{Mt_{b}}}\sum_{m=1}^{M}u_{m}\left[t-(m-1)t_{b}\right]$$

where

(15) $$u_{m}(t)=\{\begin{array}{ll} \exp(j2\pi f_{m}t), & 0\leq t\leq t_{b}\\ 0 & \mathrm{elsewhere} \end{array}$$

and

(16) $$f_{m}=a_{m}\Delta f$$

**Figure 3:**
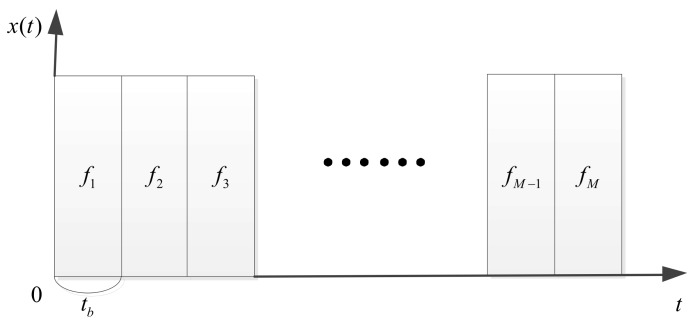
The sketch map of the IPFC signal.

Additionally, M is the total number of the sub-pulse, m is the *m*-th sub-pulse, $t_{b}$ is the sub-pulse repeat interval, $f_{m}$ denotes the frequency of the *m*-th sub-pulse, Δf denotes the frequency step between sub-pulses and $u_{m}(t)$ presents the envelope of the *m*-th sub-pulse. The sketch map of the IPFC signal is shown in Figure 3.

In general, there is always a relationship between $t_{b}$ and Δf, which can be described as

(17) $$\Delta f\leq \frac{1}{t_{b}}$$

**Figure 4:**
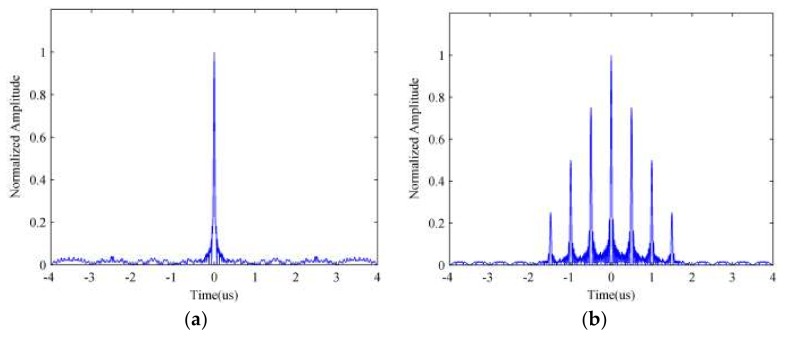
The pulse compression result of random signal with different parameters. Sub-pulse duration satisfies (**a**) $t_{b}=\frac{1}{\Delta f}$; (**b**) $t_{b}>\frac{1}{\Delta f}$.

Otherwise there would be a gap between sub-pulses in the frequency spectrum, which means that the signal can’t overspread the whole spectrum. Thus, the compression result would contain a mass of side-lobes, which would affect the target detection performance. The pulse compression results for a random signal are depicted in Figure 4a when $t_{b}=\frac{1}{\Delta f}$ and Figure 4b when $t_{b}>\frac{1}{\Delta f}$, from which we can find that the Figure 4b has plenty of side-lobes, which are distributed symmetrically on both sides of the central peak, compared with Figure 4a. Thus, the detection performance of the random frequency coded signal when $t_{b}>\frac{1}{\Delta f}$ would be seriously attenuated.

**Figure 5:**
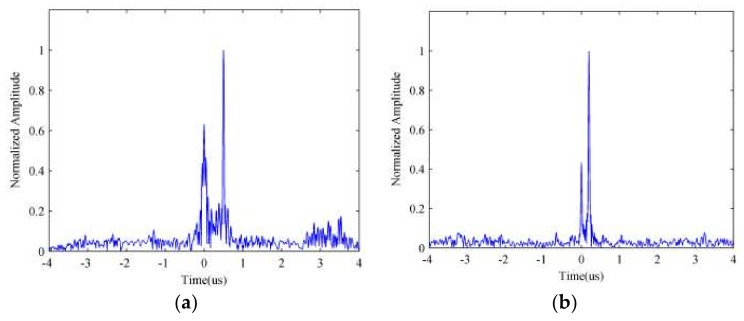
The pulse compression result of the radar echo signal containing ISRJ with different parameters transmitting random signals. Sub-pulse duration satisfies (**a**) $t_{b}=T_{s}=\frac{1}{\Delta f}$; (**b**) $T_{s}<t_{b}<\frac{1}{\Delta f}$.

According to [13], we know that the ISRJ of random signal through the matched filter can always form a single false target that delays τ behind the real target because of the uncorrelation between the jamming and the impulse response of the matched filter, which is mainly caused by the random frequency code. Moreover, as for random signal, it is unnecessary to meet the condition between the sub-pulse duration and pulse repeat interval, namely,

(18) $$t_{b}\leq T_{s}$$

which is rather critical in an intra-pulse frequency coded signal design to assure that uncorrelation and enhance the ECCM performance for ISRJ. Thus, we consider it an advantage for the random signal, even when $t_{b}>T_{s}$, whose ISRJ signal only forms a single false target through the matched filter. The pulse compression result of the radar echo signal containing the ISRJ is depicted in Figure 5a when $t_{b}=T_{s}=\frac{1}{\Delta f}$ and Figure 5b when $T_{s}<t_{b}<\frac{1}{\Delta f}$, respectively. Compared with Figure 5a, the energy of false target in Figure 5b is much stronger, because of the repeated sampling on each sub-pulse.

Thus, a parameter-adjusted random frequency coded signal, which is an optimal waveform to counter the ISRJ, is chosen as the transmitted radar signal. However, there is still a single false target at a specific location. In order to suppress the ISRJ completely, we will propose two ECCM approaches to remove the ISRJ-based central false target. In Approach I, we design a band-pass filter in the time domain to remove that central false target from the output of the matched filter. By performing Approach I, the power of the central false target can effectively be repressed, but the false target can’t be totally removed, and the values of the jamming signal ratio (JSR) and the signal-to-noise ratio (SNR) affect the ECCM performance significantly. Thus, we propose Approach II, which mainly focuses on the signal processing operation for the radar echo signal before the matched filter. By eliminating the echo in the jamming zone and splicing the rest of them together, we can retain the true target and suppress the ISRJ.

### 3.2. ECCM Approach I: Generating the Band-Pass Filtering Function

Based on the TF analysis result obtained in Section 2 and the design of the parameter-adjusted radar signal, we propose an approach to remove the single false target by generating the band pass filter. The key point in Approach I is the reconstruction of the interrupted sampled signal, whose compression result is the impulse response of the band pass filter.

(19) $$H(t)={\hat{x}}_{s}(t)⊗x^{*}(-t)$$

After multiplying the compression result of the original radar echo signal $y_{out}(t)$ with the band pass filtering impulse response. The ISRJ can ultimately be suppressed.

(20) $$y_{I}\_out=y_{out}(t)\times H(t)$$

The advantage of ECCM Approach I is that it is efficient and available even when the jammer adopts the periodic repeater method. By performing Approach I, the power of the central false target can be repressed effectively, but the false target can’t be totally removed, because of the side-lobes of the impulse response of the band pass filter, which is the compression result of the reconstructed interrupted sampled signal. Moreover, the values of JSR and SNR affect the performance of the proposed approach significantly.

### 3.3. ECCM Approach II: Signal Processing Method

To overcome the shortcomings of Approach I, Approach II is proposed at the cost of sacrificing the signal-to-noise ratio (SNR), and is mainly focused on the input of the matched filter by shearing and splicing the radar echo. The execution measures of the Approach II are denoted as follows.

Firstly, we transmit a parameter-adjusted signal, the same as with Approach I. Then, we receive the radar echo signal comprised of jammer echo signal, ISRJ signal and noise. Based on the TF analysis in Section 2, we remove the signal in the jamming zone by multiplying the radar echo signal with the reconstructed interrupted sampling function $\hat{p}(t)$, namely

(21) $$y_{R}^{\prime }(t)=y_{R}(t)\cdot \hat{p}(t)$$

Next, before matched filtering, we splice the rest of the radar echo signal into a continuous signal $y_{R}^{\prime \prime }(t)$ in the time domain. Subsequently, in order to forming the corresponding matched filter, we do the same signal processing operations on radar transmitted signal, namely, multiplying the radar transmitted signal with the reconstructed interrupted sampling function $\hat{p}(t)$

(22) $$x^{\prime }(t)=x(t)\cdot \hat{p}(t)$$

and splicing the rest of the transmitted signal denoted as $x^{\prime \prime }(t)$. The impulse response of the matched filter can thus be obtained by taking the mirror image of the conjugate of the spliced signal $x^{\prime \prime }(t)$, which can be expressed as

(23) $$H^{\prime }(t)={\left[x^{\prime \prime }(-t)\right]}^{*}$$

Ultimately, through the matched filter, the true target signal can be retained, and the central false target is suppressed. The suppression result can be expressed as

(24) $$y_{II}\_out=y_{R}^{\prime \prime }(t)⊗H^{\prime }(t)$$

The advantage and disadvantage of Approach II is explicitly. Because the jamming signals in the jamming zone are totally removed, the compression result always has a single peak belonging to the real target. However, the echo signals of the real target in the jamming zone are also removed, and the SNR of the compression result would be decreased. Therefore, when the duration of jamming zone which is decided by the sampling duty cycle and repeater times and can be expressed as $T_{j}=\tau \cdot N_{s}$ increases, the SNR of the compression result will decrease. Thus, the performance of approach II will decline seriously.

### 3.4. ECCM Approach Selection

**Figure 6:**
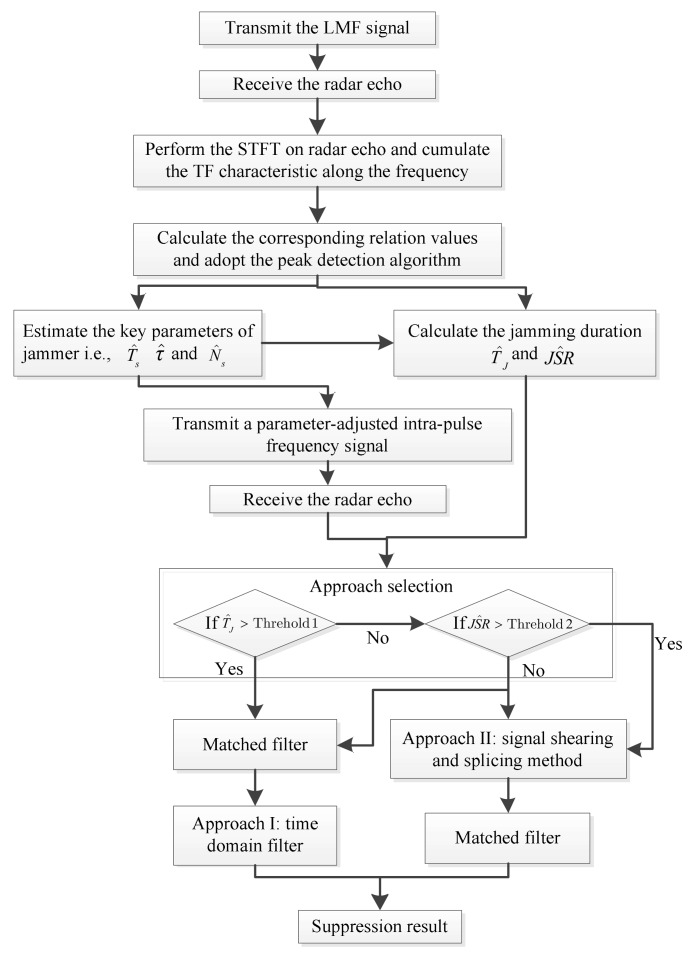
Flow chart of the proposed ECCM scheme.

As the above analysis of two proposed ECCM approaches demonstrates, both of them have advantages and disadvantages in difference scenarios. There is thus a trade-off relationship between two approaches, i.e., when jamming duration is relatively long, Approach I can be chosen. Compared with Approach I, Approach II has better performance when the jamming duration is shorter and JSR is larger. In practice, we always chose the two approaches by setting the appropriate thresholds of jamming duration and JSR in different scenarios. The specific selection procedure can be seen in Figure 6, which is the framework of the whole ECCM scheme.

## 4. Simulation

In this section, we perform computer simulations of three aspects. First, some results when we perform the ISRJ estimation process by transmitting the LMF signal based on TF analysis are presented. Then, a parameter-adjusted random signal is designed that only forms a single false target. Based on the estimated ISRJ parameters through the TF analysis and the designed radar signal, two approaches are proposed to remove the single false target, and some related simulation results are carried out to verify the performance and effectiveness of the proposed ECCM scheme. The key employed parameters are illustrated in Table 1.

### 4.1. ISRJ Parameter Estimation Results

We transmit the LMF signal to estimate the ISRJ parameters by performing a TF transform on the received echo signal. Figure 6 shows the STFT results for the received echo signal.

**Figure 7:**
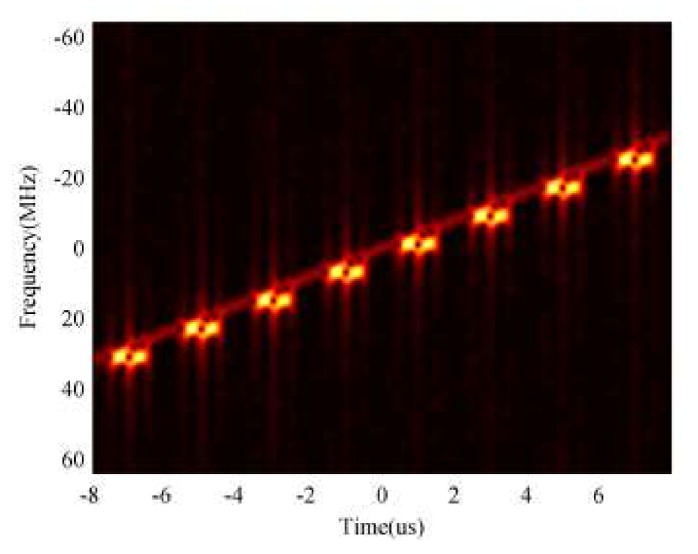
STFT results of LMF echo signal.

**Figure 8:**
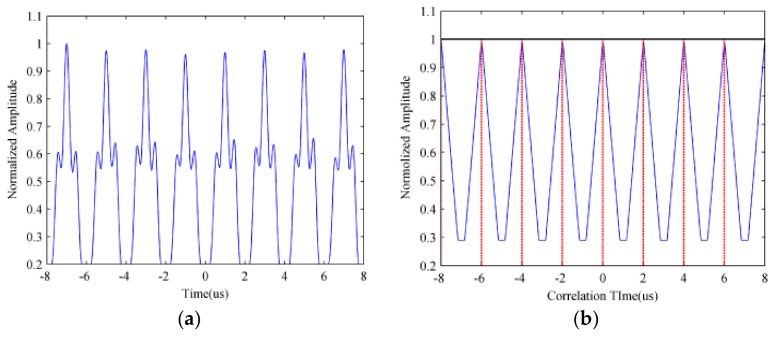
Estimation result by applying correlation detection. (**a**) The summation of the STFT along the frequency; (**b**) the autocorrelation result of STFT summation.

As shown in Figure 7, the continuous and dim straight line denotes the real target signal and the discontinuous and bright segment presents the ISRJ signal, from which we can evidently find that the TF characteristics of the received echo signal are discontinuous, as a result of ISRJ. Figure 8 gives some estimation results by applying correlation detection.

Figure 8a illustrates the summation results of the STFT along the frequency, namely S(t), which represents the periodic characteristics of the received echo signal. Figure 8b shows the autocorrelation results of S(t), and we can see that there are eight significant peaks, which indicates that the repeat sampling time is 8. Thus, we utilize the peak detection algorithm to calculate the value of two adjacent peaks’ interval, which is the estimation value of the jammer repeat sampling interval ${\hat{T}}_{s}$.

**Figure 9:**
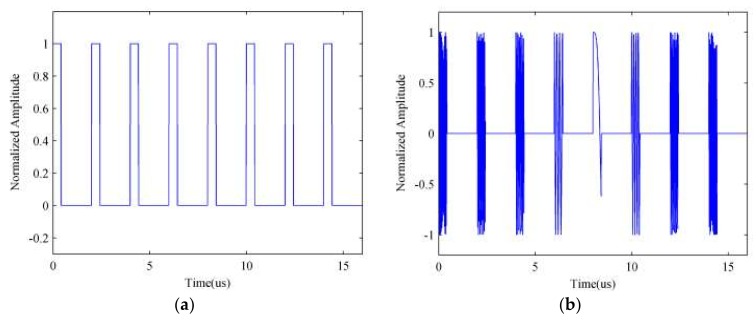
Simulation results of reconstructed signals. (**a**) Rectangular sampling sequence; (**b**) the interrupted sampled radar signal.

Based on the estimated sampling time, we can reconstruct the sampled signal ${\hat{x}}_{s}(t)$ precisely. The reconstructed sampling sequence and sampled radar signal are shown in Figure 9.

**Figure 10:**
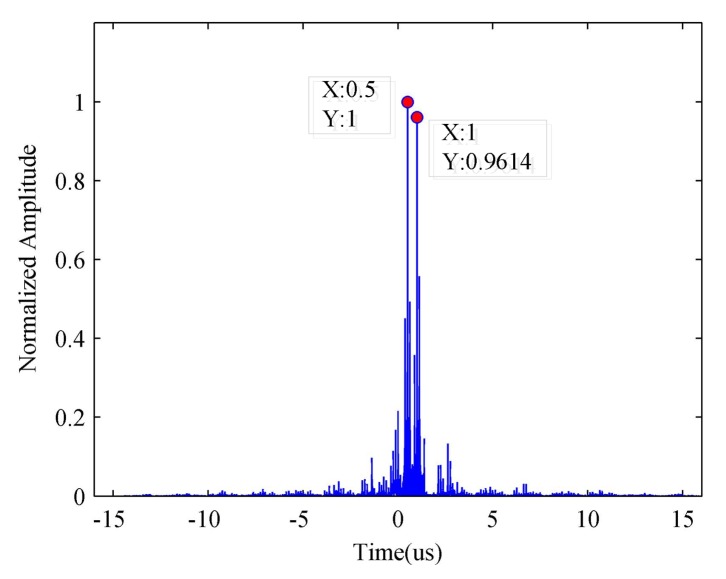
The cross-correlation result of the reconstructed sampled signal ${\hat{x}}_{s}(t)$ and radar echo signal $x_{r}(t)$.

We apply the cross-correlation operation on the reconstructed sampled signal ${\hat{x}}_{s}(t)$ and radar echo signal $x_{r}(t)$, and Figure 10 shows the cross-correlation result. By calculating the number of extreme points and any two extreme points’ interval, we thus obtained the estimated values of repeater times ${\hat{N}}_{s}$ and sampling duration $\hat{\tau }$.

**Figure 11:**
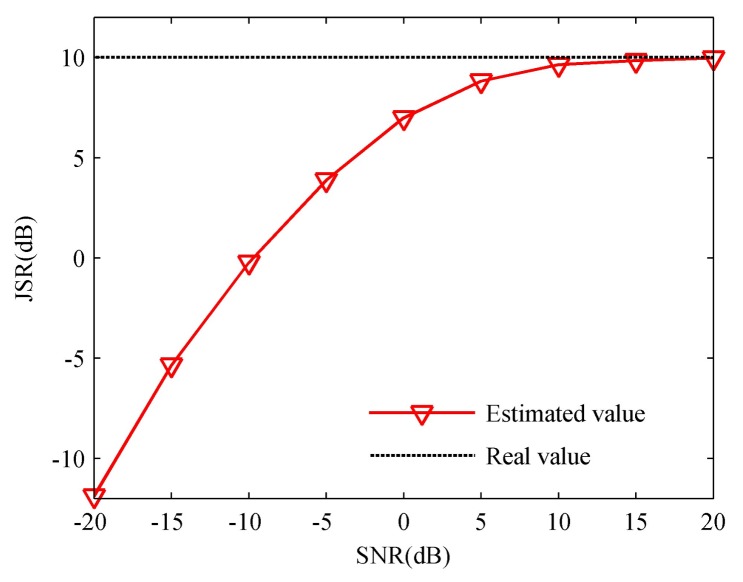
The estimated JSR versus the input of SNR.

Based on the estimated ISRJ parameters and the received radar signal, we can calculate the JSR and duty cycle of the jammer by using (11) and (12). However, in (11), we dismiss the effect of the noise when calculating the JSR. Figure 11 illustrates the estimated JSR versus the input of the SNR.

### 4.2. Simulations of Proposed ECCM Scheme for ISRJ

**Figure 12:**
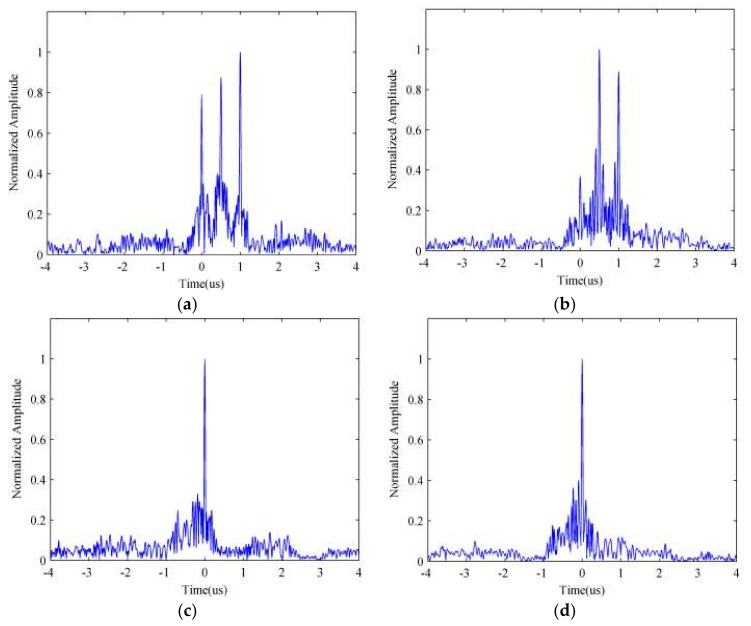

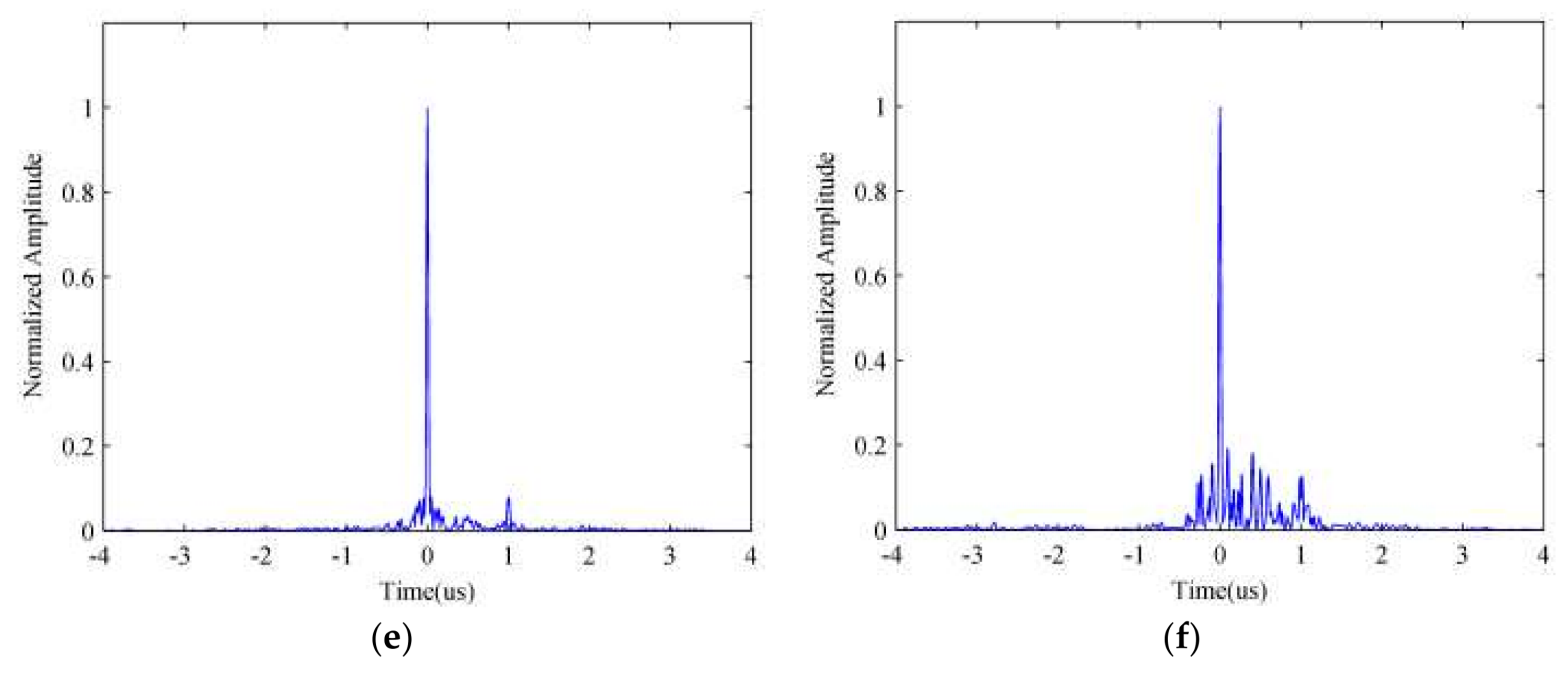
Simulation results of Approach I under different JSR conditions. (**a**) The compression result of the received signal with JSR = 10 dB; (**b**) the compression result of the received signal with JSR = 20 dB; (**c**) the band pass filtering function in the time domain with JSR = 10 dB; (**d**) the band pass filtering function in the time domain with JSR = 20 dB; (**e**) the suppression result with JSR = 10 dB; (**f**) the suppression result with JSR = 20 dB.

Based on the estimated ISRJ parameters, a parameter-adjusted intra-pulse frequency coded signal is chosen as the transmitted signal, and Approach I is applied to suppress the single false target at the output of the matched filter. The corresponding simulation results are presented in Figure 12. In Figure 12, we illustrate the compression result of the received signal containing ISRJ, the designed band pass filtering function in the time domain and the suppression results under two different JSR.

**Figure 13:**
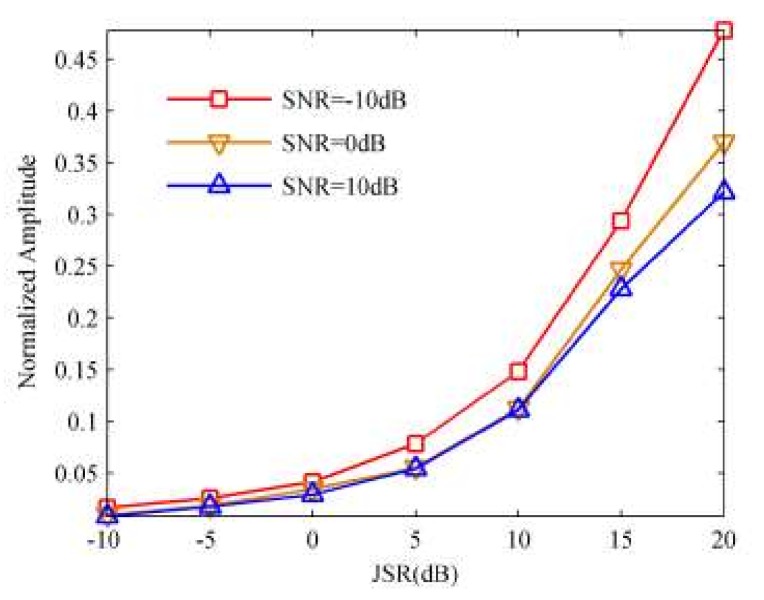
The normalized amplitude of suppressed false target versus the JSR in different SNR values.

From Figure 12, we can find that Approach I can fairly suppress the ISRJ and retain the real target, especially under low JSR. However, because the side-lobes of the band pass filtering function in the time domain, which is the compression results of the reconstructed interrupted sampled signal, the ISRJ signal lagging τ behind the real target can’t be removed completely. By comparing Figure 12f with Figure 12e, we can see that the jamming suppression result with JSR = 10 dB is better than that with JSR = 20 dB, which seems to indicate that the performance of Approach I will attenuate as the JSR increases. Further, in order to validate this phenomenon, we carry out the Monte Carlo simulation, whose repeat times are 20 under varying JSRs. Figure 13 depicts the comparative amplitude of suppressed false target versus the JSR in different SNR values, which reflects the trend of the suppression performance.

From Figure 13, we can find that the normalized amplitude of the central false target rises with the increasing of the JSR when the SNR value is constant, and with the decreasing of the SNR when JSR value is constant. We can thus draw a conclusion that the performance of Approach I is available only if the JSR is a rather small value (i.e., smaller than 10 dB) and the value of SNR is comparatively large.

**Figure 14:**
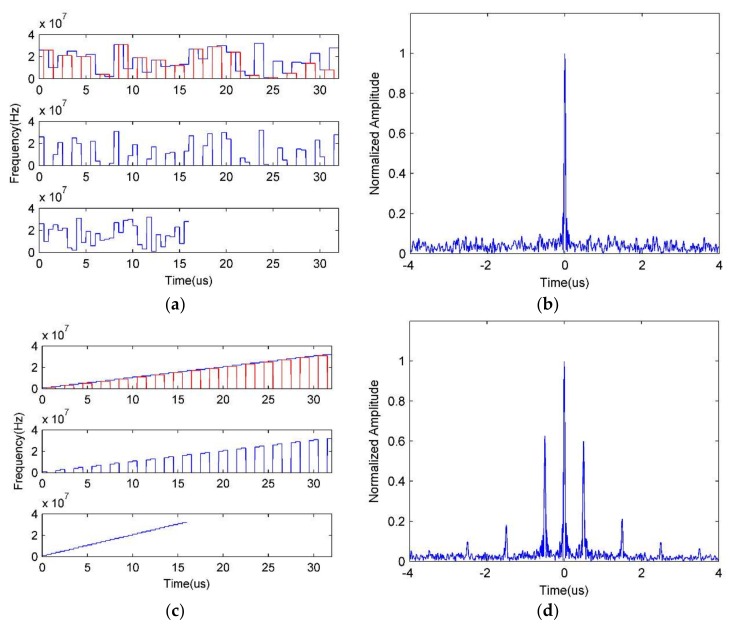
Simulation results of Approach II. (**a**) TF curves in different signal processing moments under IPFC signal; (**b**) the ISRJ suppression result under IPFC signal; (**c**) TF curves in different signal processing moments under stepped-frequency signal; (**d**) the ISRJ suppression result under stepped-frequency signal.

Thus, we tend to perform Approach II to suppress the false target after transmitting a parameter-adjusted waveform the same as with Approach I, and the related simulation results are presented in Figure 14.

Figure 14 gives the time-frequency curves and suppression results of Approach II after pulse compression. Figure 14a, from the top downward, depicts the TF curve when in a different signal processing moment, which represents the processing flow of a received radar echo signal, where the top one denotes the TF curve of the radar echo signal and the red parts represent the jamming zone; the middle one represents the radar echo signal whose jamming zone signals are removed; and the bottom one describes the spliced signal, whose pulse duration and bandwidth have been contracted, after the signals in the jamming zone have been removed. Figure 14b shows the suppression results of Approach II. We can find that the false target has been completely suppressed, and the ECCM performance is acceptable even when JSR is large because the jamming signals have been totally removed.

In order to highlight the effectiveness of Approach II under IPFC signal, we also present the results under stepped-frequency signal (which is similar to LFM signal). As shown in Figure 14c,d, we can see that since the frequency continuity of target echo signal is destroyed, the pulse compression result will suffer from high side-lobes, which will decrease the target detection performance. Therefore, it can be concluded that the IPFC signal with Approach II has a preferable performance against ISJR.

**Figure 15:**
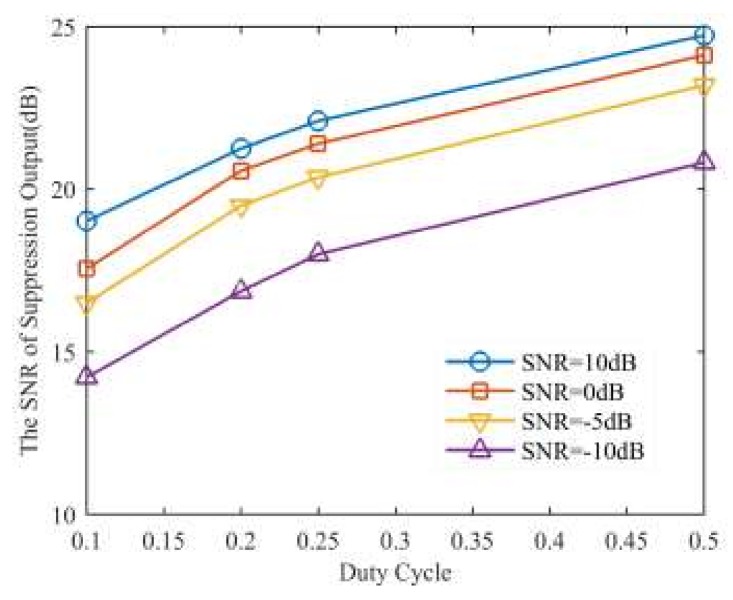
The SNR of suppression results versus duty cycle under different SNRs.

According to the derivation of Approach II, we know that the SNR of suppression result will be decreased, which will affect the ECCM performance significantly. In Figure 15, we analyze the efficiency of Approach II in different SNR and duty cycles, which corresponds to the jamming duration when the jammer only moves forward once, namely, $N_{s}(t)=1$. As shown in Figure 14, the SNR of suppression results decreases as duty cycle rises, and with the increasing of SNR, the SNR of the suppression results will increase accordingly.

## 5. Conclusions

In this paper, based on the design of a parameter-adjusted intra-pulse signal and the estimated parameters of a jammer through TF analysis, two effective ECCM approaches are proposed to remove the ISRJ-based false targets from the pulse compression result. The executing steps of the ECCM scheme are presented, in which the particular band-pass filter is generated, and the signal processing methods are adopted. Detailed simulations prove the significant ECCM performance of the proposed scheme.

However, it should be stated that the proposed scheme cannot be directly applied to situations with multi-targets or multi-jammers. In multi-jammer cases, it is difficult to estimate the ISRJ parameters due to the jamming signals’ interlaced overlap. In this case, we should try to separate the jamming signals first, and then apply the proposed ECCM scheme separately. Thus, this paper is only a preliminary one, and how to counter the ISRJ in a complicated scenario is a perspective which will be investigated in our future research work.

## Figures and Tables

**Table 1:** Simulation parameters.

Parameter	Value	Unit
Bandwidth of LMF signal B	64	MHz
Pulse width of LMF signal $T_{P}$	16	us
Radar Sampling frequency fs	128	MHz
Jammer Sampling duration τ	0.5	us
Jammer Repeat Sampling Interval $T_{s}$	2	us
Repeater times $N_{s}$	2	
Jamming to signal ratio JSR	10	dB
Signal to noise ratio *SNR*	10	dB
Bandwidth of random signal $B^{\prime }$	32	MHz
Pulse width of random signal $T_{P}^{\prime }$	32	us
Sub-pulse number M	32	

## References

[1] Mahafza B.R., Elsherbeni A.Z. (2004). Matlab Simulations for Radar Systems Design.

[2] Neri F. (2006). Introduction to Electronic Defense Systems.

[3] Schleher D.C. (1999). Electronic Warefare in the Information Age.

[4] Wang X.S., Liu J.C., Zhang W.M. (2007). Mathematic principles of interrupted-sampling repeater jamming. Sci. China Ser. F Inform. Sci..

[5] Liu P., Bao Q.L., Chen Z.P. (2007). Design of False Target Deception Jammer against PD Radar. Mod. Radar.

[6] Feng D.J., Tao H.M., Yang Y. (2011). Jamming de-chirping radar using interrupted-sampling repeater. Sci. China Inform. Sci..

[7] Gong S.X., Wei X.Z., Li X. (2014). ECCM Scheme against Interrupted Sampling Repeater Jammer Based on Time-Frequency Analysis. J. Syst. Eng. Electron..

[8] Liu Q., Xing S., Wang X., Feng D.J. (2012). The interferometry phase of InSAR coherent jamming with arbitrary waveform modulation. Prog. Electromagn. Res..

[9] Jiang Y., He M.H., Liu H.B., Yu C.L. (2016). Recognition of interrupted-sampling repeater jamming based on resemblance coefficient. Mod. Radar.

[10] Zhou C., Tang Z.Y., Fu F.L., Lu Y.X. (2017). Anti intermittent sampling repeater jamming method based on intrapulse orthogonality. J. Syst. Eng. Electron..

[11] Xu L.T., Feng D.J., Zhang W.M. (2012). Effectiveness analysis of jamming phase-coded signal using interrupted-sampling repeater. Electron. Warf..

[12] Levanon N. (2004). Radar Signals. Hoboken.

[13] Shen R., Liu Z., Sui J.P., Wei X.Z. Study on Interrupted-Sampling Repeater Jamming Performance Based on Intra-Pulse Frequency-Coded Signal. Proceedings of the 2016 Electornic Warefare National Conference.

[14] Cohen L. (1995). Time-Frequency Analysis. Englewood Cliffs.

[15] Chen C.V., Hao L. (2002). Time-Frequency Transforms for Radar Imaging and Signal Analysis.

